# Editorial: Interactive effects of plant growth-promoting microbes and nanoparticles on the physiology, growth, and yield of crops

**DOI:** 10.3389/fpls.2024.1338470

**Published:** 2024-03-07

**Authors:** Aqeel Ahmad, Waheed Akram, Rehana Sardar, Nasim Ahmad Yasin

**Affiliations:** ^1^ Institute of Geographic Sciences and Natural Resources Research / University of Chinese Academy of Sciences, CAS, Beijing, China; ^2^ Department of Plant Pathology, Faculty of Agricultural Sciences, University of the Punjab, Lahore, Pakistan; ^3^ Institute of Botany, University of the Punjab, Lahore, Pakistan; ^4^ RO-II Office, University of the Punjab, Lahore, Pakistan

**Keywords:** PGPM, NPS, plant nutrition, plant growth, sustainable crop production

Continuous demand of plant-based nutritious food from the ever-growing population has become an alarming issue throughout the world. Inorganic, organic and hybrid nanoparticles (NPs) having 1-100 nm size have enormous potential to improve crop yield and assure sustainability. Nanoparticles are essential for plant development and quality enhancement because they boost photosynthetic activity, improve metabolism, and increase nutritional content. Yield and quality of agricultural crops may be enhanced through the judicious application of nano techniques which increases effectiveness of inputs and reduces related losses. Nanotechnology assists in preserving stability of pathogen-free food items. Nanomaterials have been used to develop pesticides, fertilizers and other plant growth promoting products. An enormous quantity of various fertilizers is required for the improvement of soil fertility leading to higher crop yield. However, the recurrently applied higher quantity of fertilizers negatively affects soil health and fertility. A large quantity of conventional fertilizer persists in the rhizosphere or may leach down causing environmental contamination that injures the adjacent fauna and flora. Hence, the appropriate administration of fertilizer is a tricky task for the development of sustainable agriculture. Nano fertilizers not only mitigate nutrient deficiency but also release nutrients according to the needs of the crop. Therefore, higher fertilizer use efficiency by applying nano fertilizers enables farmers to obtain better crop yield without polluting the ecosystem.

Plant growth-promoting microbes (PGPM) may improve plant stress tolerance, fertilizer use efficiency and nutrient uptake potential of assisted plants. Such microbes live in plant tissues or within rhizospheric area and sustain plant growth by several mechanisms including enhancement of hormonal synthesis, phytostimulation, augmentation of stress tolerance besides improvement of nutrients availability and uptake. Hence, application of PGPM should be examined to decrease the dependance on synthetic agrochemicals to enhance crop yield. *In vitro* studies, under controlled and ideal environments, have shown beneficial effects of PGPM inoculation on various crops. Nevertheless, further field experimentations especially under environmental extremes seems mandatory to evaluate the effects of plant microbe cross talk to advance growth and yield of inoculated plants.

Keeping in view the importance of nanoparticles and PGPM, the Guest Editors of the Research Topic made a call for research manuscripts through Frontiers in Plant Science. Guest Editors encouraged scientists to submit their manuscript regarding crucial aspects related to beneficial effects of PGPM and NPs on the physiology, growth, and yield of crop plants. It is expected that this Research Topic will be very productive to trigger a quest in researchers to find the dynamic potential of NPs and PGPM to increase plant growth. Research articles for this Research Topic come from various continents. Scientists applied numerous physiological, biochemical, molecular procedures to elucidate the interaction among NPs, PGPM and plants.

In this Research Topic 27 manuscripts were received. However only 10 manuscripts having the better quality were accepted for publication following peer-review. Adoko et al. found that PGPR-based bio stimulants combined with mineral fertilizer enhanced growth, yield and nutritional status of maize. Ahmad et al. reported that differentially expressed genes of *Cymbidium ensifolium*, *C. goeringii* and *C. sinense* were mainly related to bacterial secretion systems (*FLS2, CNGCs* and *EFR*) which adjust hypersensitive response, stomatal movement and defense induction. Ali et al. revealed that growth promoting *Bacillus* strain NMTD17 improves the activity of antioxidative enzymes and stress tolerance of rice plants under saline conditions. Mirza et al. demonstrated that application of bio-fabricated graphene oxide nanoparticles enhances growth and seed yield of *Vigna radiata*.

Sehar et al. found that combined application of *Serendipita indica* and phosphorus alleviate the arsenic stress in rice through genotype-dependent modulations. Microbes may enhance the medicinal value of associated plants. Zhang et al. observed the role of *Cercospora* inoculation on growth and active ingredients of *Polygonum hydropiper* sp. Sohail and Chen proposed a systematic approach on assessment of sustainable agriculture in climate change particularly in floods. Zhou et al. concluded that intercropping improved soil health, microbial community, plant growth and biomass production in tobacco. Salehi et al. concluded that manganese nanoparticles improved seed germination, photosynthetic activity, antioxidative activity and plant growth in *Artemisia annua.* Jalal et al. observed synergistic effects of nano-Zn and *B. subtilis* and *Pseudomonas fluorescens* in improving wheat plant growth and nutrition.

In general, the manuscripts selected for this Research Topic divulge the importance of interaction among NPs, PGPM and plants. However, further studies at the transcriptomics, metabolomics, proteomics and genomics level are required to identify and demonstrate beneficial and harmful interactions among NPs, PGPM and plants to continue sustainable crop production under normal and harsh environmental conditions. Conclusively, this Research Topic includes valuable research articles which enables scientists and community workers to acquire the basic information about NPs, PGPM and plant communication.

## Author contributions

AA: Conceptualization, Writing – review & editing. WA: Conceptualization, Writing – original draft, Writing – review & editing. NY: Conceptualization, Writing – original draft, Writing – review & editing. RS: Validation, Writing – review & editing.

